# Resveratrol Ameliorates Aortic Calcification in Ovariectomized Rats via SIRT1 Signaling

**DOI:** 10.3390/cimb43020075

**Published:** 2021-09-05

**Authors:** Sally K. Hammad, Rana G. Eissa, Mohamed A. Shaheen, Nahla N. Younis

**Affiliations:** 1 Department of Biochemistry, Faculty of Pharmacy, Zagazig University, Zagazig 44519, Egypt; ranaeissa@hotmail.com (R.G.E.); nahlayounis2003@yahoo.com (N.N.Y.); 2 Department of Histology and Cell Biology, Faculty of Medicine, Zagazig University, Zagazig 44519, Egypt; drmohamedshaheen@yahoo.com

**Keywords:** menopause, vascular calcification, estrogen, phytoestrogen, resveratrol, polyphenol, SIRT1

## Abstract

Postmenopausal women are at an increased risk of vascular calcification which is defined as the pathological deposition of minerals in the vasculature, and is strongly linked with increased cardiovascular disease risk. Since estrogen-replacement therapy is associated with increased cancer risk, there is a strong need for safer therapeutic approaches. In this study we aimed to investigate the protective and therapeutic effects of the phytoestrogen resveratrol against vascular calcification in ovariectomized rats, a preclinical model of postmenopause. Furthermore, we aimed to compare the effects of resveratrol to those of estrogen and to explore the mechanisms underpinning those effects. Treatment with resveratrol or estrogen ameliorated aortic calcification in ovariectomized rats, as shown by reduced calcium deposition in the arterial wall. Mechanistically, the effects of resveratrol and estrogen were mediated via the activation of SIRT1 signaling. SIRT1 protein expression was downregulated in the aortas of ovariectomized rats, and upregulated in rats treated with resveratrol or estrogen. Moreover, resveratrol and estrogen reduced the levels of the osteogenic markers: runt-related transcription factor 2 (RUNX2), osteocalcin and alkaline phosphatase (ALP) which have been shown to play a role during vascular calcification. Additionally, the senescence markers (p53, p16 and p21) which were also reported to play a role in the pathogenesis of vascular calcification, were reduced upon treatment with resveratrol and estrogen. In conclusion, the phytoestrogen resveratrol may be a safer alternative to estrogen, as a therapeutic approach against the progression of vascular calcification during postmenopause.

## 1. Introduction

Natural menopause is defined as twelve consecutive months of amenorrhea that are not the result of other causes [1]. The median age at which natural menopause occurs is fifty years old [2]. The life expectancy for women is more than eighty years, in at least thirty-five countries, including the US. This means that women will spend up to thirty years of their lives as postmenopausal [1,3,4].

During menopausal transition and postmenopause, which are characterized by a decline in estrogen, women experience several symptoms. These symptoms include vasomotor and psychological symptoms, sleep disturbances, in addition to genitourinary and sexual function changes. Postmenopausal women also have increased risk of cognitive disturbances, osteoporosis, vascular calcification and cardiovascular disease [1,5,6,7,8,9,10,11].

Vascular calcification is defined as the pathological deposition of mineral in the vasculature and is strongly linked with increased cardiovascular disease risk [12,13]. Studies have shown that postmenopausal women are at a greater risk of aortic calcification and cardiovascular disease [1,9,11].

The increased cardiovascular risk in postmenopausal women has been linked to the declining levels of endogenous estrogen. Data from the Framingham study showed that cardiovascular disease incidence in postmenopausal women was 2 to 6 times higher compared to premenopausal women of the same age [1,14]. The protective effects of estrogen before menopause explain at least in part the fact that on average cardiovascular disease develops in women a decade later than men [15].

Clinical trials have shown that estrogen-replacement therapy can have beneficial effects against arterial calcification and cardiovascular disease outcomes and mortality. These beneficial effects are seen when estrogen-replacement therapy is initiated in women who are younger than sixty years, or less than ten years after menopause. On the other hand, when estrogen-replacement therapy is initiated at an older age or more than ten years after menopause, no protective effects are seen and harmful effects may occur [1].

Importantly, estrogen-replacement therapy has been associated with increased risk of venous thromboembolism, stroke, and different kinds of cancer including breast, ovarian and endometrial cancer [16,17,18]. Therefore, there is a strong need for safer therapeutic approaches that provide postmenopausal women with protective effects against arterial calcification and cardiovascular disease risk.

Resveratrol belongs to a group of compounds of plant origin, known as phytoestrogens. Due to their structural similarity to estrogen, phytoestrogens can bind estrogen receptors and produce similar effects. Moreover, evidence suggests their use is related to lowered cancer risk, including breast and ovarian cancers. Therefore, phytoestrogens such as resveratrol have been suggested as a safer alternative to estrogen [19,20]. Resveratrol (3,4′,5-trihydroxy-stilbene) is a polyphenolic compound that naturally occurs in grapes, peanuts, berries and legumes. Interestingly, an in vitro study showed that resveratrol had a protective effect against vascular calcification in rat aortic smooth muscle cells, an effect that seemed to be mediated via Sirtuin 1 (SIRT1) activation [21,22].

SIRT1 is a key member of sirtuins, which is a family of highly conserved NAD^+^ dependent deacetylases. Polymorphisms in SIRT1 gene have been associated with coronary artery calcification, abnormalities in cholesterol metabolism, and early-onset coronary artery disease [23,24]. Although SIRT1 downregulation has been shown to enhance calcification of rat aortic smooth muscle cells in vitro, [21], the underlying mechanisms remain to be elucidated. Moreover, the potential in vivo effects of resveratrol against vascular calcification during postmenopause remain to be investigated.

In addition to SIRT1, the transdifferentiation of vascular smooth muscle cells (VSMCs) into osteoblast-like cells has been implicated in vascular calcification pathogenesis. The osteogenic transcription factor runt-related transcription factor 2 (RUNX2), and the osteogenic markers osteocalcin and alkaline phosphatase (ALP) have been shown to play a role during vascular calcification [25,26,27]. Additionally, in vitro studies have linked the osteogenic differentiation of VSMCs to an increased expression of the senescence markers p53, p21 and p16 [28,29].

A myriad of studies has investigated the role of osteoprotegerin (OPG) and receptor activator of nuclear factor-κB ligand (RANKL) in bone. Their role in the vasculature, however, has received less attention. In vitro evidence suggests that RANKL may increase the calcification of VSMCs, particularly when OPG is inhibited. On the other hand, OPG may play a protective role against vascular calcification [30,31,32,33].

As far as we know, no previous studies have explored the potential protective effects of resveratrol against vascular calcification, compared them to those of estrogen or investigated the underlying mechanisms in ovariectomized rats, a preclinical model of postmenopause. Thus, this study aimed to investigate the protective and therapeutic effects of resveratrol against vascular calcification in ovariectomized rats. Moreover, it aimed to compare the effects of resveratrol to those of estrogen and to explore the mechanisms underpinning those effects.

## 2. Materials and Methods

### 2.1. Drugs

Resveratrol (Res) was purchased from Mega Resveratrol (Danbury, CT, USA) and estradiol valerate (E2) was supplied by Chemical Industries Development Co. (CID) (Cairo, Egypt).

### 2.2. Animals and Ovariectomy

Female rats (200–220 g) were bought from the National Research Centre (Giza, Egypt). Rats were kept under controlled conditions of temperature, humidity and 12 h light–dark cycle in the animal unit at Zagazig University. Animal care, experimental design and procedures were in accordance with the guidelines of the National Institute of Health (NIH) and were approved by Zagazig University-Institutional Animal Care and Use Committee (ZU-IACUC).

Rats were allowed one week to acclimatize, after which they were allocated to ovariectomized (OVX) and sham groups. To induce postmenopause, rats in the ovariectomized groups were anesthetized and underwent a bilateral removal of ovaries, as previously described [34]. A similar procedure was performed in sham rats, without the removal of ovaries.

### 2.3. Study Design

Eight weeks following the surgery (ovariectomy or sham), rats were allocated to 4 groups: (1) Sham group; (2) Ovariectomized group (OVX); (3) Estradiol group (E2): ovariectomized rats that were treated with estradiol valerate 0.8 mg/kg/day, orally for 8 weeks [35] and (4) Resveratrol group (Res): ovariectomized rats that were treated with resveratrol 80 mg/kg/day, orally for 8 weeks [35].

### 2.4. Sampling

At the end of the study, rats were sacrificed, and their aortas were collected. The aortas were washed in ice-cold phosphate-buffered saline and periaortic fat was removed. Aortas were snap-frozen in liquid nitrogen then stored at −80 °C until required for further analysis. Histology samples were immersed in 4% paraformaldehyde solution till further processing [36].

### 2.5. Western Blot Analysis

To extract proteins from aorta samples, they were homogenized in RIPA buffer containing protease inhibitors and the homogenates were centrifuged at 12,000 rpm for 10 min at 4 °C. Total protein concentration in each sample was quantified using Bradford assay (Bio Basic, Markham, ON, Canada). Protein samples were aliquoted and stored −80 °C until required for further analysis.

Polyacrylamide gel electrophoresis was used to separate the protein samples, which were then transferred to polyvinylidene fluoride membranes. Additionally, 3% bovine serum albumin in tris-buffered saline with Tween 20 (TBST) was used to block the membranes for one hour at room temperature. Membranes were incubated with SIRT1 primary antibody (Santa Cruz Biotechnology, Dallas, TX, USA), overnight at 4 °C, then washed in TBST, and incubated with HRP-conjugated secondary antibody (Santa Cruz Biotechnology, Dallas, TX, USA). The membranes were washed in TBST and Clarity™ Western ECL Substrate (Bio-Rad, Hercules, CA, USA) was used to detect protein bands. Beta actin (a housekeeping protein) was used to normalize the band intensity of the target protein.

### 2.6. Enzyme-Linked Immunosorbent Assay (ELISA)

ELISA kits were used to determine runt-related transcription factor-2 (RUNX-2) (Aviva Systems Biology, San Diego, CA, USA), osteocalcin (Novus Biologicals, Centennial, CO, USA), alkaline phosphatase (ALP) (BioVision Incorporated, Milpitas, CA, USA), osteoprotegerin (OPG) (LifeSpan Biosciences Inc., Seattle, WA, USA), receptor activator of nuclear factor-κB ligand (RANKL) (Biorbyt Ltd., Cambridge, UK) and tumor protein p53 (p53) (Novus Biologicals, Centennial, CO, USA) in aorta samples. The manufacturers’ instructions were followed.

### 2.7. Reverse Transcription Polymerase Chain Reaction (RT-PCR)

RNA was extracted from aorta samples using TRIzol (Invitrogen, Waltham, MA, USA), following the manufacturer’s instructions. RNA pellets were resuspended in nuclease-free water. RNA quantity and quality were assessed using a NanoDrop spectrophotometer (ThermoFisher Scientific, Waltham, MA, USA). RNA was reverse transcribed to complimentary DNA (cDNA) using High-Capacity cDNA Reverse Transcription Kit (Applied Biosystems, Waltham, MA, USA), following the manufacturer’s protocol [36]. The quantitative polymerase chain reactions were performed using SensiFAST™ SYBR^®^ Lo-ROX Kit (Bioline, London, UK) [29]. The expression of target genes (p21 and p16) was normalized to that of the housekeeping gene, β-actin using the 2^-∆∆Ct^ method [29,36]. Primers sequence for p21 gene was the following: forward 5′-TGTTCCACACAGGAGCAAAG-3′ and reverse 5′-AACACGCTCCCAGACGTAGT-3′, for p16 gene was; forward 5′-CATCTCCGAGAGGAAGGCGAACT-3′ and reverse 5′-CGCAGTTCGAATCTGCACCATAG-3′, and β-actin housekeeping gene was; forward 5’-CTAAGGCCAACCGTGAAAAG-3’ and reverse 5’-GCCTGGATGGCTACGTACA-3’.

### 2.8. Histological Study

Aortic tissue was fixed in 4% paraformaldehyde solution, embedded in paraffin, then a microtome was used to produce 5 μm thick sections. Sections were dewaxed with xylene and hydrated before they were stained with hematoxylin and eosin (H&E). H&E-stained sections were used to measure aortic intima media thickness. For Alizarin Red S staining, sections were dehydrated, placed in an Alizarin Red S solution, then rinsed in distilled water to remove any unbound stain. Alizarin Red stained-sections were used to evaluate aortic calcification [36,37,38]. Images were analyzed using ImageJ 1.53c software (National Institutes of Health, Bethesda, MD, USA).

### 2.9. Statistical Analysis

Statistical analysis was performed using Graphpad Prism 8 software (GraphPad Software, San Diego, CA, USA). Results are expressed as mean ± SD. One-way analysis of variance (ANOVA) and Tukey’s post-hoc test were used to assess the statistical difference between groups. A *p*-value of less than 0.05 was considered statistically significant.

## 3. Results

### 3.1. Resveratrol and E2 Upregulated SIRT1 Expression in the Aortas of Ovariectomized Rats

**Figure 1:**
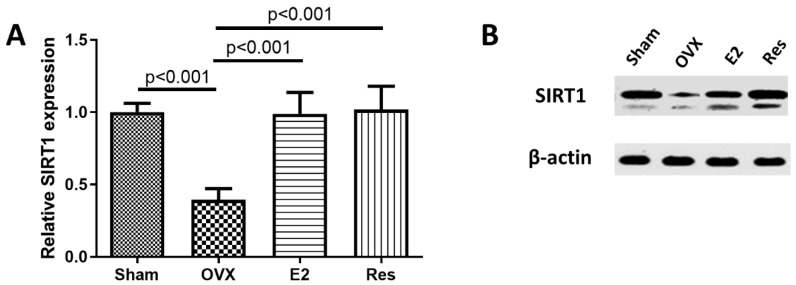
Resveratrol and E2 upregulated SIRT1 protein expression in the aortas of ovariectomized rats. (**A**) Relative SIRT1 protein expression, normalized to β-Actin (arbitrary units). (**B**) Representative Western blot images. OVX: ovariectomized rats; E2: estradiol; Res: resveratrol. Graphs show mean ± SD, *n* = 6.

SIRT1 gene polymorphisms have been associated with arterial calcification and SIRT1 downregulation has been shown to enhance the calcification of rat aortic smooth muscle cells *in vitro* [21,23,24,39]. Therefore, we investigated the effects of ovariectomy as a postmenopausal model, and treatment with resveratrol or E2 on SIRT1 protein expression in the aorta. SIRT1 protein expression was significantly downregulated in the aortas of OVX rats, compared to rats in the sham group (*p* < 0.001). Treatment of OVX rats with either resveratrol or E2 significantly upregulated SIRT1 protein expression, compared to untreated OVX rats (*p* < 0.001), Figure 1.

### 3.2. Resveratrol and E2 Reduced the Aortic Levels of Osteogenic Markers

**Figure 2:**
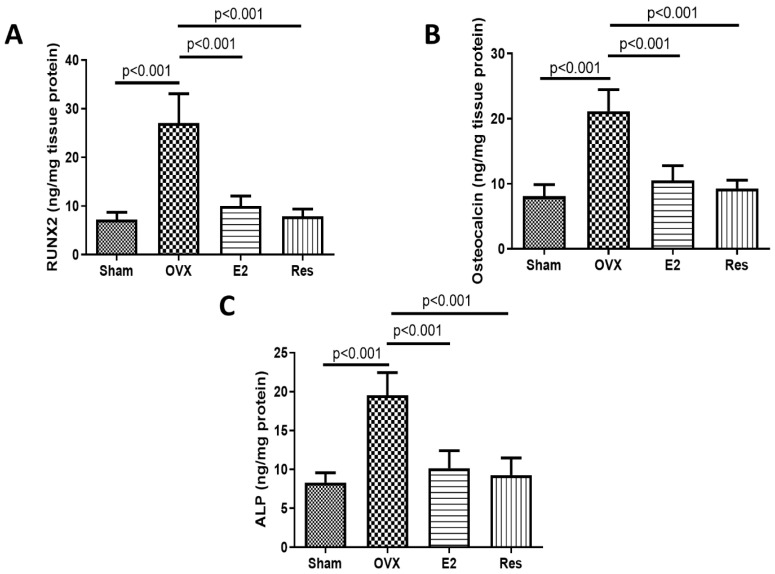
Resveratrol and E2 reduced the aortic levels of osteogenic markers. (**A**) RUNX2 level. (**B**) Osteocalcin level (**C**) ALP level. Protein levels were quantified using ELISA. OVX: ovariectomized rats; E2: estradiol; Res: resveratrol. Graphs show mean ± SD, *n* = 6.

The osteogenic transdifferentiation of VSMCs into osteoblast-like cells has been implicated in vascular calcification pathogenesis [25,26,27]. Therefore, we investigated the effects of ovariectomy and treatment with resveratrol and E2 on the aortic levels of RUNX2, a key osteogenic transcription factor, and the osteogenic markers osteocalcin and ALP. The levels of RUNX2, osteocalcin and ALP were significantly increased in the aortas of OVX rats, compared to sham (*p* < 0.001). Resveratrol and E2 significantly reduced the aortic levels of these osteogenic markers, compared to untreated OVX rats (*p* < 0.001), Figure 2.

### 3.3. Resveratrol and E2 Significantly Increased OPG and Decreased RANKL Aortic Levels

**Figure 3:**
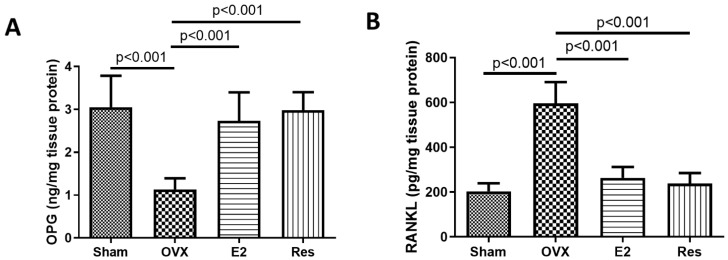
Resveratrol and E2 significantly increased OPG and decreased RANKL aortic levels. The protein levels of (**A**) OPG and (**B**) RANKL. Protein levels were quantified using ELISA. OVX: ovariectomized rats; E2: estradiol; Res: resveratrol. Graphs show mean ± SD, *n* = 6.

Although the role of OPG and RANKL in the vasculature has been studied less extensively than their role in bone, in vitro evidence suggests that RANKL may increase vascular calcification, particularly when OPG is inhibited. On the other hand, OPG may play a protective role against vascular calcification [30,31,32,33]. In our study, the aortas of OVX rats showed a significant decrease in OPG and a significant increase in RANKL, compared to sham rats (*p* < 0.001). Resveratrol and E2 significantly increased OPG and decreased RANKL aortic levels, compared to the OVX group (*p* < 0.001), Figure 3.

### 3.4. Resveratrol and E2 Downregulated the Expression of the Senescence Markers p53, p21 and p16 in the Aortas of OVX Rats

**Figure 4:**
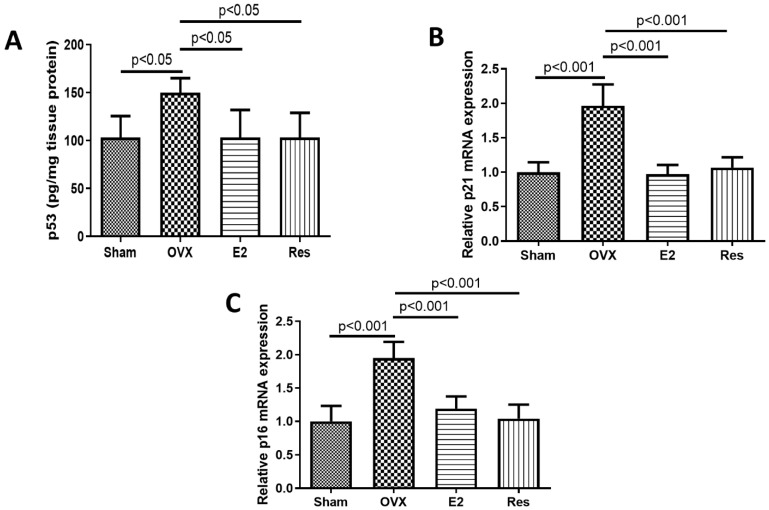
Resveratrol and E2 downregulated the expression of the senescence markers p53, p21 and p16 in the aortas of OVX rats. (**A**) p53 protein level in aorta. (**B**) Relative p21 mRNA expression in aorta. (**C**) Relative p16 mRNA expression in aorta. p53 protein levels were quantified using ELISA. p21 and p16 mRNA expression levels were quantified using RT-PCR. OVX: ovariectomized rats; E2: estradiol; Res: resveratrol. Graphs show mean ± SD, *n* = 6 each.

The osteogenic differentiation of VSMCs has been linked to an increased senescent capacity in vitro [28,29]. In our study, the aortas of OVX rats showed a significant increase in the osteogenic markers: RUNX2, osteocalcin and ALP. Therefore, we investigated whether senescence plays a role in arterial calcification during postmenopause. The expression of the senescence markers p53, p21 and p16 was significantly upregulated in the aortas of OVX rats, compared to sham. Compared to the OVX group, resveratrol and E2 treatment significantly downregulated p53 expression (*p* < 0.05), and the expression of p21 and p16 (*p* < 0.001), Figure 4.

### 3.5. Resveratrol and E2 Improved Aortic Calcification in OVX Rats

**Figure 5:**
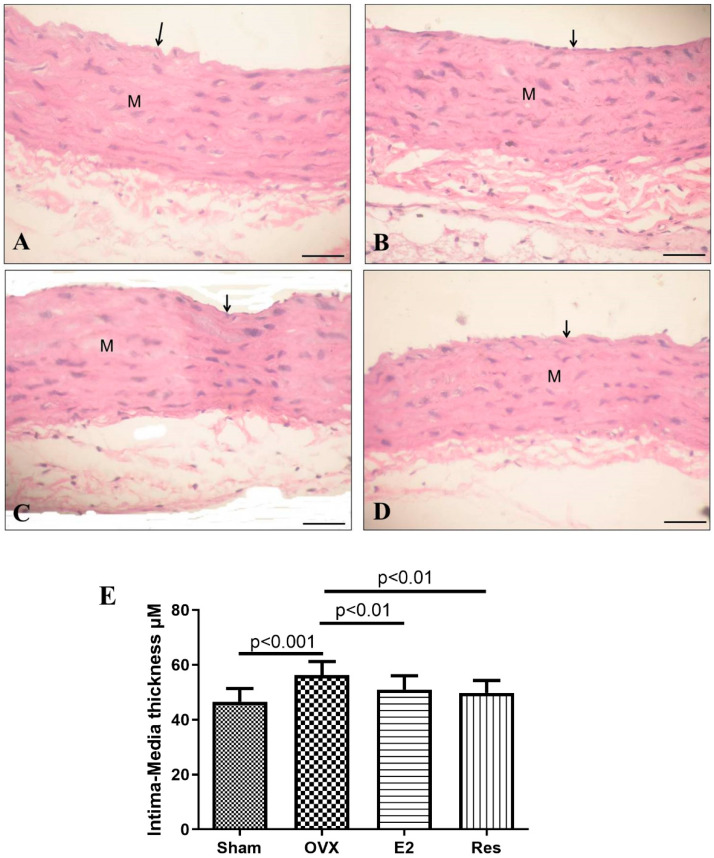
Resveratrol and E2 improved aortic intima media thickness, as shown by H&E stain. (**A**) The sham group showing a tunica intima that consists of a simple squamous epithelium (arrow) lining the interior surface of the vessel with an intact internal elastic lamina. A normal subendothelial layer of loose connective tissue is also shown. The tunica media (M) is made of smooth muscle cells and elastic fibers. (**B**) OVX rats showing signs of intimal injury as denoted by the separation of the endothelial layer. (**C**) Estradiol-treated rats and (**D**) resveratrol-treated rats. (**E**) Aortic intima media thickness. Graph shows mean ± SD. Scale bar 20 μm. OVX: ovariectomized rats; E2: estradiol; Res: resveratrol.

Histopathological examination of H&E-stained aortic sections from the sham group showed that the tunica intima consisted of simple squamous epithelium lining the interior surface of the vessel with an intact internal elastic lamina. Moreover, it showed a normal subendothelial layer of loose connective tissue. The tunica media was made of smooth muscle cells and elastic fibers. On the other hand, OVX rats showed signs of intimal injury. The aortic intima media thickness in OVX rats was significantly higher than the sham group (*p <* 0.001). Treatment with resveratrol or estradiol significantly reduced aortic intima media thickness, compared to OVX rats (*p* < 0.01), Figure 5.

**Figure 6:**
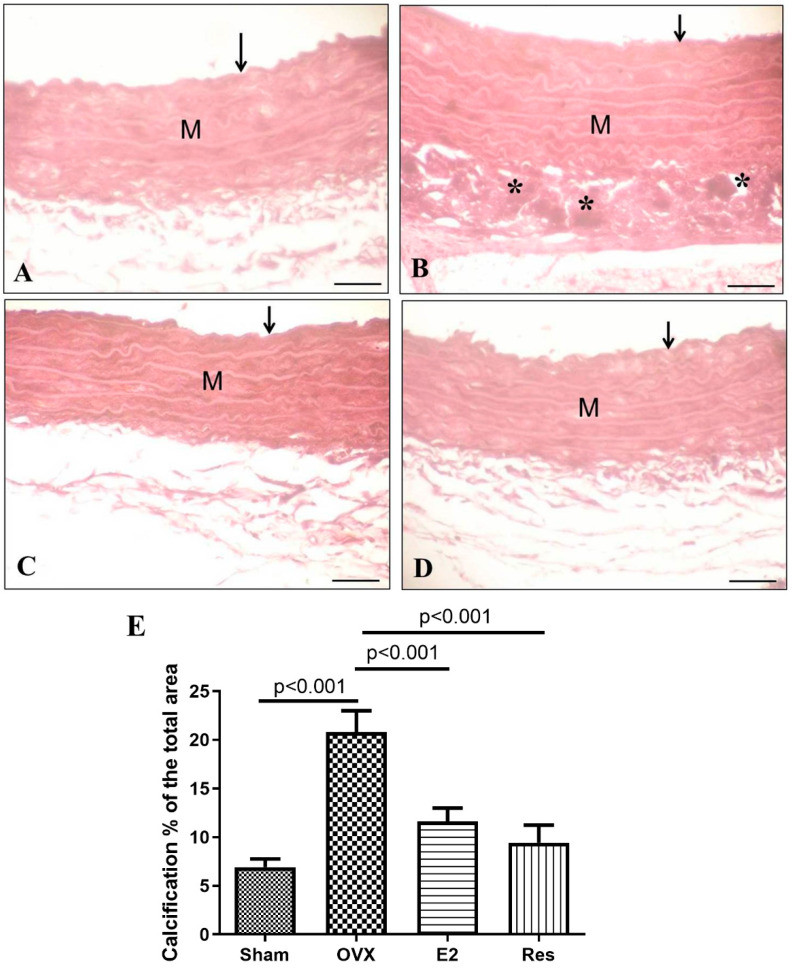
Resveratrol and estradiol ameliorated aortic calcification in ovariectomized rats, as shown by Alizarin red-stained sections. (**A**) The sham group showing tunica intima (arrow) and tunica media (M). (**B**) OVX rats showing marked calcium deposition (*) in the tunica media (M). (**C**) Estradiol-treated rats. (**D**) Resveratrol-treated rats. (**E**) Aortic calcification percentage. Graph shows mean ± SD. Scale bar 20 μm. OVX: ovariectomized rats; E2: estradiol; Res: resveratrol.

Aortic calcification was evaluated using Alizarin red-stained sections. The aortas of OVX rats showed a greater calcification burden (calcium deposition) than the sham (*p* < 0.001). Aortic calcification was significantly improved upon treatment with resveratrol or estradiol (*p* < 0.001), Figure 6.

## 4. Discussion

Estrogen deficiency caused by menopause leads to various physiological and psychological effects. In addition to disturbed sleep patterns and mood swings, low estrogen levels can lead to arterial vasoconstriction [40]. Importantly, postmenopausal women are at an increased risk of vascular calcification, which is strongly linked with increased cardiovascular disease risk [41]. Since estrogen-replacement therapy is associated with increased cancer risk, there is a strong need for safer therapeutic approaches. In our study, we assessed for the first time the therapeutic effects of the phytoestrogen, resveratrol against aortic calcification in ovariectomized rats. Furthermore, we compared the effects of resveratrol to those of estrogen and investigated the mechanisms underlying the effects of resveratrol and estrogen treatment in a preclinical postmenopausal model.

The presence of vascular calcification in the aortas of ovariectomized rats was confirmed using Alizarin red staining and established markers of VSMC osteogenic differentiation including RUNX2, osteocalcin and ALP [29,32,42,43,44]. Additionally, the expression of the senescence markers p53, p16 and p21 was increased, suggesting that they play a role in the development of vascular calcification. On the other hand, SIRT1 expression and OPG levels were reduced in untreated ovariectomized rats, while RANKL levels and aortic intima media thickness were increased. Treatment with resveratrol or estrogen ameliorated aortic calcification, downregulated the expression of osteogenic and senescence markers, increased SIRT1 expression and OPG levels and reduced RANKL and aortic intima media thickness. These findings suggest that resveratrol may be a safer alternative to estrogen, as a therapeutic approach against the progression of arterial calcification during postmenopause and that SIRT1 and OPG may play a protective role against the development of arterial calcification in the context of postmenopause.

Sirtuins are a family of highly conserved NAD^+^ dependent deacetylases, of which SIRT1 is a key member. Previous studies have associated SIRT1 gene polymorphisms with coronary artery calcification, abnormalities in cholesterol metabolism, and early-onset coronary artery disease [23,24,39]. Moreover, SIRT1 downregulation has been shown to enhance the calcification of rat aortic smooth muscle cells in vitro [21]. Therefore, we aimed to investigate whether SIRT1 is involved in the etiology of menopause-induced vascular calcification. In accordance with previous findings by Sasaki et al., we showed decreased SIRT1 levels in the aortas of the OVX group, compared to sham [45]. In vitro studies have shown that estrogen can increase SIRT1 levels in human aortic endothelial cells and vascular smooth muscle cells [46,47]. In agreement with these in vitro studies, estrogen treatment significantly increased SIRT1 levels in the aortas of ovariectomized rats. Similarly, ovariectomized rats treated with resveratrol had significantly higher SIRT1 levels than the OVX group. Our findings are in accordance with Zhang et al. and Han et al. who showed that resveratrol upregulated the expression of SIRT1 in calcified rat and human VSMCs [21,48].

Runt-related transcription factor 2 (RUNX2) is a key osteogenic transcription factor that has been shown to play a role in the transdifferentiation of VSMCs into osteoblast-like cells during vascular calcification [25,26]. SIRT1 is involved in the regulation of several transcription factors including RUNX2, with SIRT1 showing different regulatory properties depending on cell type [29]. SIRT1 suppression in vitro has been linked to the upregulation of RUNX2, the osteogenic transdifferentiation of VSMCs and the progression of vascular calcification [29]. In our study, ovariectomy led to reduced aortic SIRT1 levels. These levels were significantly improved upon treatment with estrogen or resveratrol. These changes in SIRT1 levels could explain the increased levels of RUNX2 in the OVX group, compared to sham and treatment groups which had lower RUNX2 levels. Our in vivo findings are in agreement with in vitro studies where resveratrol upregulated the expression of SIRT1 and downregulated the expression of RUNX2 in calcified rat and human VSMCs [21,48], and estrogen produced similar results in VSMCs including human aortic smooth muscle cells [31,46,47].

In addition to RUNX2, other osteogenic markers such as osteocalcin and ALP have been shown to play a role in the transdifferentiation of VSMCs during vascular calcification [25,26,27]. Osteocalcin is a non-collagenous bone matrix protein that has been strongly linked to vascular calcification. Elevated osteocalcin levels were found in the calcified arterial smooth muscle layer of diabetic patients and in calcified aortic valves [29,49,50,51]. ALP is an enzyme that plays a key role in inducing vascular calcification. It depletes pyrophosphate, which is a potent calcification inhibitor [29,49,52]. In our study, the aortas of ovariectomized rats showed increased osteocalcin and ALP levels, compared to sham. Treatment with estrogen or resveratrol significantly reduced osteocalcin and ALP levels, compared to OVX rats. Our study supports and extends previous in vitro findings showing that SIRT1 inhibition is pivotal in modulating osteogenic factors. The chemical inhibition and genetic knockdown of SIRT1 have been shown to increase osteocalcin and ALP levels, while SIRT1 activation has been shown to reduce them in VSMCs [29,53]. Since SIRT1 levels were reduced in the aortas of the OVX group and improved in the treatment groups, they could explain the changes seen in osteocalcin and ALP levels. The increased osteocalcin and ALP levels in the OVX group, which were reduced upon treatment with resveratrol or estrogen, can also be explained by the changes in RUNX2 expression which showed a similar pattern. RUNX2 has been shown to control ALP expression, and osteocalcin is one of its downstream targets [49,50,51].

The role of osteoprotegerin (OPG) and receptor activator of nuclear factor-κB ligand (RANKL) in bone has been extensively studied. Although the role of OPG and RANKL in the vasculature has been studied less extensively, evidence suggest that RANKL may increase the calcification of VSMC in vitro, particularly when OPG is inhibited. On the other hand, OPG may play a protective role against vascular calcification [30,31,32,33]. OPG knockout mice (OPG^-/-^) showed medial calcification of the aorta and renal arteries. Moreover, OPG administration significantly reduced the osteogenic transformation and calcification in the aortic valves of hypercholesterolemic mice [54,55,56].

In accordance with these findings, our results show that the calcified aortas of the ovariectomized group had lower OPG and higher RANKL levels than the sham group. Resveratrol and estrogen treatment increased OPG and reduced RANKL levels. This is in accordance with previous studies where estrogen and resveratrol produced similar results in bone [57,58]. SIRT1 activation has been shown to increase OPG expression in VSMCs, while SIRT1 inhibition decreased OPG expression [29]. Our study showed reduced SIRT1 and OPG levels in the aortas of OVX rats. These levels were improved upon treatment with resveratrol or estrogen. Based on these findings, we postulate that resveratrol and estrogen via SIRT1 activation may play a key role in modulating OPG/RANKL signaling in aortic calcification.

The osteogenic differentiation of VSMCs has been linked to an increased senescent capacity *in vitro* [28,29]. In our study, the calcified aortas of OVX rats showed a decrease in the levels of SIRT1 and an increase in the levels of osteogenic markers RUNX2, osteocalcin and ALP. Therefore, we investigated whether senescence plays a role in vascular calcification in menopause. The expression of the senescence markers p53, p21 and p16 was significantly increased in the aortas of OVX rats, compared to sham. Resveratrol and estrogen-treated rats had significantly lower expression levels of senescence markers than the OVX group. This is in accordance with an in vitro study where VSMCs treated with estrogen had lower p53 expression compared to untreated controls [47].

SIRT1, a deacetylase, has been shown to decrease p53 expression in vitro in VSMCs. This effect was attributed to a significant reduction in lysine acetylation in the p53 promotor region. Additionally, it has been reported that p53 acetylation induces cellular senescence, while p53 deacetylation abrogates it [29,59,60,61,62]. p21 is one of the key targets of p53. It is associated with DNA damage, which plays a role in the pathological regulation of calcification. The expression of both p53 and p21 was found to be significantly upregulated in vitro in VSMCs, when SIRT1 is diminished or completely inhibited [28,29,63]. Moreover, in vitro studies have shown that p16 expression is upregulated when certain sites on its promoter are acetylated by p300 or when the levels of the deacetylase SIRT1 are reduced [29,64]. Our data support and expand on these previous findings, demonstrating that SIRT1, upregulated by resveratrol or estrogen, downregulates p53, p21 and p16 which may contribute to modulating senescence and arterial calcification in OVX rats.

## 5. Conclusions

**Figure 7:**
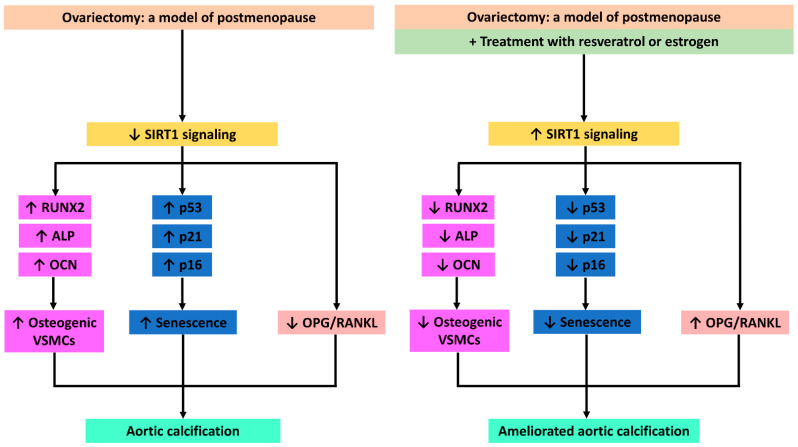
Resveratrol and estradiol ameliorated aortic calcification in ovariectomized rats via SIRT1 signaling.

Our findings suggest that SIRT1 downregulation plays a key role in arterial calcification during postmenopause (Figure 7). We showed that the phytoestrogen resveratrol ameliorated arterial calcification in ovariectomized rats, a preclinical model of postmenopause. Furthermore, resveratrol produced similar effects to those of estrogen. Those effects seem to be mediated via the activation of SIRT1 signaling, the downregulation of markers of osteogenesis (RUNX2, osteocalcin and ALP) and senescence (p53, p21 and p16), and the modulation of OPG/RANKL signaling. Since resveratrol lacks the risks associated with estrogen-replacement therapy, we suggest that it might be a safer alternative as a therapeutic approach against the progression of arterial calcification during postmenopause.

## Data Availability

The data that support the findings of this study are available from the corresponding author upon reasonable request.

## Abbreviations

ALP	Alkaline phosphatase
E2	Estradiol
H&E	Hematoxylin and eosin
OPG	Osteoprotegerin
OVX	Ovariectomized
RANKL	Receptor activator of nuclear factor-κB ligand
Res	Resveratrol
RUNX2	Runt-related transcription factor 2
SIRT1	Sirtuin 1
VSMCs	Vascular smooth muscle cells
